# Impact of Metastatic Microenvironment on Physiology and Metabolism of Small Cell Neuroendocrine Prostate Cancer Patient-Derived Xenografts

**DOI:** 10.3390/cancers17142385

**Published:** 2025-07-18

**Authors:** Shubhangi Agarwal, Deepti Upadhyay, Jinny Sun, Emilie Decavel-Bueff, Robert A. Bok, Romelyn Delos Santos, Said Al Muzhahimi, Rosalie Nolley, Jason Crane, John Kurhanewicz, Donna M. Peehl, Renuka Sriram

**Affiliations:** Department of Radiology and Biomedical Imaging, University of California, San Francisco, CA 94563, USA; shubhangi.ag@gmail.com (S.A.); jinnysun@berkeley.edu (J.S.); emiliedecavelbueff@gmail.com (E.D.-B.); robert.bok@ucsf.edu (R.A.B.); romelyn.delossantos@ucsf.edu (R.D.S.); said.almuzhahimi@ucsf.edu (S.A.M.); rosalie.nolley@ucsf.edu (R.N.); jason.crane@ucsf.edu (J.C.); john.kurhanewicz@ucsf.edu (J.K.); donna.peehl@ucsf.edu (D.M.P.)

**Keywords:** hyperpolarized carbon-13 magnetic resonance imaging, glycolysis, small cell neuroendocrine cancer, microenvironment, metastases, metabolism, physiology, perfusion

## Abstract

**Background:** Potent androgen receptor pathway inhibitors induce small cell neuroendocrine prostate cancer (SCNC), a highly aggressive subtype of metastatic androgen deprivation-resistant prostate cancer (ARPC) with limited treatment options and poor survival rates. Patients with metastases in the liver have a poor prognosis relative to those with bone metastases alone. The mechanisms that underlie the different behavior of ARPC in bone vs. liver may involve factors intrinsic to the tumor cell, tumor microenvironment, and/or systemic factors, and identifying these factors is critical to improved diagnosis and treatment of SCNC. Metabolic reprogramming is a fundamental strategy of tumor cells to colonize and proliferate in microenvironments distinct from the primary site. Understanding the metabolic plasticity of cancer cells may reveal novel approaches to imaging and treating metastases more effectively. **Methods:** Using magnetic resonance (MR) imaging and spectroscopy, we interrogated the physiological and metabolic characteristics of SCNC patient-derived xenografts (PDXs) propagated in the bone and liver, and used correlative biochemical, immunohistochemical, and transcriptomic measures to understand the biological underpinnings of the observed imaging metrics. **Results:** We found that the influence of the microenvironment on physiologic measures using MRI was variable among PDXs. However, the MR measure of glycolytic capacity in the liver using hyperpolarized ^13^C pyruvic acid recapitulated the enzyme activity (lactate dehydrogenase), cofactor (nicotinamide adenine dinucleotide), and stable isotope measures of fractional enrichment of lactate. While in the bone, the congruence of the glycolytic components was lost and potentially weighted by the interaction of cancer cells with osteoclasts/osteoblasts. **Conclusion:** While there was little impact of microenvironmental factors on metabolism, the physiological measures (cellularity and perfusion) are highly variable and necessitate the use of combined hyperpolarized ^13^C MRI and multiparametric (anatomic, diffusion-, and perfusion- weighted) ^1^H MRI to better characterize pre-treatment tumor characteristics, which will be crucial to evaluate treatment response.

## Simple Summary

Powerful drugs targeting androgen receptors can trigger a very aggressive form of metastatic prostate cancer known as small cell neuroendocrine prostate cancer (SCNC). This type of cancer is tough to treat and has a poor survival rate, especially when it spreads to the liver, compared to when it spreads only to the bones. The objective was to understand why SCNC tumors behave differently in the liver versus the bones, which is critical for improving how we diagnose and treat this severe cancer using imaging. To this end, the same SCNC cells derived from patients were implanted in mouse bone and liver, and many different parameters were investigated using magnetic resonance technology. The results showed that the metabolic activity of cancer cells in the liver matched certain biochemical markers, whereas in the bones, these markers were influenced by interactions with bone cells. These findings suggest that the tumor’s environment significantly affects its characteristics, and hence, it is essential to use a combination of imaging methods to accurately assess the tumor before treatment. This approach could lead to better treatment strategies and improve the evaluation of how well treatments are working. By understanding these mechanisms, researchers can develop new ways to image and treat cancer, potentially leading to more effective therapies for patients with metastatic prostate cancer. This research is crucial for advancing cancer treatment and could have a significant impact on the medical community’s approach to managing aggressive cancer types.

## 1. Introduction

Small cell neuroendocrine prostate cancer (SCNC) is an aggressive subtype of prostate cancer (PCa) defined by characteristic features such as a high nuclear to cytoplasmic ratio and indistinct cell borders [1], low or no expression of the androgen receptor (AR) [2], and poor prognosis [3]. SCNC is rare in primary PCa [4], whereas about 10–17% of patients with metastatic androgen deprivation-resistant prostate cancer (ARPC) have been reported to develop treatment-related SCNC (t-SCNC) after receiving second-generation androgen receptor pathway inhibitors (ARPIs) [5]. The increase in t-SCNC is believed to be caused by heightened therapeutic pressure on the AR signaling pathway [6]. For instance, Abida et al. reported an increase in SCNC in biopsies from patients with metastatic ARPC who had undergone androgen deprivation (ADT) (10.5%) compared to those who had not (2.3%) [7]. Moreover, an increase in the incidence of SCNC was also reported following the approval of abiraterone and enzalutamide, rising from 6.3% in 1998–2011 to 13.3% in 2012–2016 [8].

The diagnosis and treatment of SCNC are challenging. Standard imaging approaches, such as ^18^F-fluorodeoxyglucose (^18^F-FDG) positron emission tomography (PET), thus far do not reliably distinguish SCNC from the more common adenocarcinomas [9] and SCNC is typically negative on a prostate-specific membrane antigen (PSMA) PET [10]. Platinum-based therapy is the mainstay treatment for SCNC, but resistance quickly develops, with no consensus on the approach for second-line therapies [11]. Molecular characterization of patient-derived tumors has revealed key players in the development of SCNC that may lead to novel targeted therapies [12,13]. Furthermore, the availability of patient-derived xenografts (PDXs) from SCNC has enabled functional studies that have enhanced understanding of this unique subtype of PCa [14]. For example, comparison of multiple SCNC PDXs revealed that some responded better to chemotherapy while others were more sensitive to targeted therapy [15]. These findings suggest that SCNC may respond differently to therapy, which might also be influenced by the site of metastasis, highlighting the need to study PDXs in clinically relevant sites.

Dysregulated metabolism is a hallmark of cancer that is relevant to the development of new imaging and therapeutic approaches. Metabolic reprogramming has emerged as a key component in the transition of adenocarcinoma to SCNC [16]. Furthermore, the tumor microenvironment at each metastatic site provides a unique niche that may modulate tumor metabolism and provide unique metabolic therapeutic vulnerabilities [17]. The major site of PCa metastases is bone; while metastases to the liver are less frequent, they are associated with a worse prognosis and poor response to therapy [18]. The effect of the bone microenvironment on prostatic adenocarcinomas has been extensively characterized [19], while interactions between the liver and prostate cancer are less understood [20]. Few studies have yet investigated interactions between SCNC and bone or liver, which are likely to differ considerably from those between adenocarcinomas and bone or liver.

Here, we selected three SCNC PDXs and evaluated engraftment, growth, and immunohistology after implantation under the renal capsule (an optimal and commonly used site of serial propagation of xenografts) and in bone and liver to replicate clinically relevant metastatic sites. The architectural characteristics of the PDX were assessed using a comprehensive multiparametric magnetic resonance imaging (MRI) approach that included T_2_-weighted anatomical imaging, diffusion-weighted imaging (DWI), and dynamic contrast-enhanced (DCE) imaging. Steady-state levels and the flux of metabolites were identified by high-field nuclear resonance spectroscopy (NMR) of extracts of [U-^13^C]glucose-labeled tumors, and glycolytic activity was evaluated using hyperpolarized (HP) [1-^13^C]pyruvate MRI. Additionally, the use of HP ^13^C-urea provided an independent measure of perfusion. RNA-Seq analysis was used to further characterize the transcriptomic differences that may arise as a consequence of the tumor site of growth. Our results provide a comprehensive profile of the metabolic and imaging characteristics of SCNC PDXs, as well as insights into the effects of the microenvironment on the SCNC phenotype that are relevant to developing new approaches to diagnose and treat SCNC.

## 2. Materials and Methods

### 2.1. Animal Models

The SCNC PDX LuCaP 93 [21] originated from the University of Washington, and LTL352 and LTL610 [22] (Living Tumour Laboratory) were developed at the Vancouver Prostate Centre. All animal studies were conducted as per the policies of the Institutional Animal Care and Use Committee (IACUC) at the University of California, San Francisco (UCSF). Both LTL610 and LuCaP 93 were established from tissues obtained using transurethral resection of the prostate, while LTL352 was established from tissue obtained from the urethra. Six- to eight-week-old male NOD SCID gamma (NOD.Cg-*Prkdc^scid^ Il2rg^tm1Wjl^*/SzJ) mice were purchased from Jackson Laboratories. The subrenal capsule (SRC) site was chosen for the propagation of tumors for further implantation into the tibia and liver to assess microenvironmental influences. Tumor tissues were implanted under the SRC following the steps described in the protocol in [23]. Mice were scanned regularly by ^1^H MRI to monitor tumor growth. When the tumors reached an optimal size (~1 cc), they were harvested, digested into single cells, and cryopreserved as described in [24]. Freshly isolated or thawed cells were injected into the tibia and liver (1 × 10^6^ viable cells in 20 μL of buffer per site) as described in [25,26].

### 2.2. Short Tandem Repeat (STR) Profiling

Frozen tumor tissues were sent to the University of California, Berkeley, DNA sequencing facility or the Genetic Resources Core Facility at the Johns Hopkins University for the extraction of DNA and STR analysis using a GenePrint 10 kit. Profile search was performed against the repositories ATCC and DSMZ.

### 2.3. ^1^H MRI

MRI was conducted using a 3 Tesla (T) horizontal scanner (BioSpec 105 mm bore diameter, Bruker) (Billerica, MA, USA) equipped with a dual-tuned ^1^H-^13^C volume coil. Mice were anesthetized with 1–2% inhalant isoflurane. A tail vein catheter was placed for venous access. T_2_-weighted images (RARE, echo time [TE] = 45 ms, repetition time [TR] = 2 s, number of excitations [NEX] = 8) with a matrix size of 192 × 192, field of view (FOV) of 32 × 32 mm, and slice thickness of 1 mm, were acquired weekly to monitor tumor growth with a ^1^H quadrature volume coil. ^1^H diffusion-weighted images were also acquired using the following parameters: matrix size, 96 × 96; FOV, 32 × 32 mm; slice thickness, 1 mm; 7 *b*-values ranging between 11 and 600 s/mm^2^.

Dynamic contrast-enhanced (DCE) measurements of intrahepatic and intratibial tumors were performed to determine the concentration of Magnevist in tumors. Baseline T_1_-weighted images were acquired using a gradient echo sequence with 3ms TE, 40 ms TR, flip angle (FA) of 40 degrees, FOV 32 × 32 mm, and 1 mm slice thickness. A total of 5 slices with a 0.5 mm gap were acquired. Baseline T_1_ was measured using a spin-echo-based saturation recovery sequence with TE = 8.25 ms, 10 variable TRs ranging from 369.5 ms to 5 s, 2 NEX, with the same FOV, slice thickness, and number of slices as T_1_-weighted images. T_1_ maps were generated using the Bruker software Paravision 6.0. Magnevist was then injected intravenously, and T_1_-weighted images were acquired every 3 s for one minute, followed by an image every minute for a total duration of 8 min. The contrast agent concentration at each time point was then estimated on a voxel-by-voxel basis by converting the intensity to concentrations of the gadolinium contrast agent using the known plasma relaxivity of Magnevist at 3 T of 3.8 mM^−1^ s^−1^ [27] and calculated T_1_ maps as before [28].

### 2.4. ^13^C HP MRI

A HyperSense™ DNP polarizer (Oxford Instruments, Abingdon, UK) was used to polarize the ^13^C probes as described previously [29]. Twenty-four microliters of neat [1-^13^C] pyruvic acid (Isotec Stable Isotopes, Miamisburg, OH, USA) with 16.5 mM trityl radical [tris(8-carboxy-2,2,6,6,-tetra(methoxyethyl)benzo [1,2-d:4,5-d′]bis(1,3)dithiole-4-yl)methyl sodium salt] (GE Healthcare, Waukesha, WI, USA) and 1.5 mM Dotarem^®^ (Bayer, Whippany, NJ, USA), and 55 µL of ^13^C-urea [6.4 M in glycerol, (Isotec Stable Isotopes, Miamisburg, OH, USA) with 17.5 mM trityl radical OX63 (Oxford Instruments, Abingdon, UK) and 0.2 mM Dotarem^®^ were co-polarized. Optimum polarization was achieved by adding the urea and pyruvic acid solutions to a sample cup and freezing them rapidly in a liquid nitrogen bath to form two separate glass layers, as previously described [30]. This was followed by dissolution in 4.5 mL of buffer containing 40 mM Tris, 80 mM NaOH, and 0.3 mM Na_2_EDTA. The dissolution mixture contained 80 mM [1-^13^C] pyruvic acid and 74 mM ^13^C-urea, with an average pH of 7.0 ± 0.5.

All experiments were performed on a preclinical 3T cryogen-free Bruker Biospin (Billerica, MA, USA) with a maximum gradient strength of 960 mT/m and a maximum slew rate of 3550 T/m/s. A dual-tuned Bruker 40 mm 1H/13C volume coil was used to acquire dynamic images using spectrally and spatially selective echo planar imaging 10 s post-intravenous infusion of 350 μL of hyperpolarized neutralized 80 mM [1-^13^C]pyruvate solution as before [29]. Briefly, a 2D chemical shift imaging pulse sequence used the following parameters: slice thickness of 8 mm, FOV 32 × 32 mm, matrix 8 × 8, flip 10° was used to acquire spectra every 4.2 s with a spectral width of 2 KHz and 128 points for a total of 15 time points.

### 2.5. MRI Data Analysis

Tumor volumes were calculated by manually segmenting the tumor on T_2_-weighted images. Tumor doubling time was then calculated as the natural logarithm of 2 divided by the slope of the linearized growth curve (using log-transformed volume) for each animal using PRISM (GraphPad, Boston, MA, USA). The ^1^H diffusion-weighted images were used to generate ADC (apparent diffusion coefficient) maps using mono-exponential fitting (*S_b_/S*_0_ = *e*(−*b* × *ADC*)), where *S_b_* is the signal intensity at a given *b* value and *S*_0_ *is* the signal intensity for *b* = 0 s/mm^2^. The mean tumor ^1^H ADC was calculated from the same region of interest (ROI) used for tumor segmentation to measure volume. HP spectroscopic images were pre-processed using the open-source SIVIC package [31] to generate metabolic images of [1-^13^C] lactate, [1-^13^C] pyruvate, and ^13^C-urea, and subsequently used for quantitative analysis using MATLAB (Release 2019a, Mathworks, Nattick, MA, USA). The same ROIs used for tumor segmentation were then overlaid on the corresponding HP metabolite images of [1-^13^C] lactate and [1-^13^C] pyruvate as before [29] to generate the apparent pyruvate to lactate conversion rate, k_PL_, using inputless single compartment unidirectional fit and expressed as a mean of the whole tumor [32]. HP ^13^C-urea images were represented as a mean tumor value, urea_AUC_, by integrating the area under the dynamic curve and normalized to blood vessel values. Only voxels with metabolite signal-to-noise ratios ≥3 were considered in the analyses.

DCE data were analyzed as before [30] and are presented as the quantitative parameter K^trans^ (volume transfer constant), which represents the v_e_ (fractional volume of the extracellular extravascular space) and v_p_ (the volume fraction) permeability using the Tofts model.

### 2.6. Stable Isotope Resolved Metabolomics (SIRM) of PDX Tumors

Metabolic labeling of PDX tumors and subsequent processing of tissue and analysis with high-resolution NMR were performed identically to Sun et al. [33]. Briefly, after HP imaging, the mice were injected with 80 μL of 25% wt/vol [U-^13^C]glucose into the tail vein every 15 min thrice under anesthesia. Tissue collected immediately after 45 min was flash-frozen in liquid nitrogen and subsequently extracted using 1:1:1 methanol–water–chloroform. The aqueous fraction isolated was measured on an 800 MHz Bruker Avance I (Bruker, Billerica, MA, USA) equipped with a 5 mm triple resonance TXI cryoprobe, using high-resolution 1D proton and 2D homo and heteronuclear sequences. Metabolomic data analysis was performed using Chenomx (Chenomx Inc., Edmonton, AB, Canada) for the assignment of peaks, and a combination of Mesternova and Topspin (Bruker BioSpin, Billerica, MA, USA) for quantification. Fractional enrichment (FE) and concentration of metabolites were computed.

### 2.7. Biological Correlates

Following HP MRI, mice were euthanized, and the tumors were rapidly dissected. A portion of each tumor was immediately fixed in 10% buffered formalin for immunohistochemical staining. The rest was snap-frozen and stored at −80 °C for subsequent biochemical assays. Tissues were homogenized using a TissueLyser (Qiagen, Germantown, MD, USA) in cell lysis buffer (Cell Signaling Technology, Danvers, MA, USA). As previously described, a colorimetric assay was used to analyze lactate dehydrogenase (LDH) activity [34]. A Lineweaver–Burke plot was used to determine the maximum velocity (Vmax) normalized to the amount of protein detected using a Bradford protein assay (Bio-Rad Laboratories, Hercules, CA, USA). The NAD+/NADH ratio was determined using a colorimetric kit (BioVision Incorporated, Milpitas, CA, USA) and normalized to the amount of protein (determined by a Bradford protein assay).

### 2.8. Immunohistochemistry (IHC)

Formalin-fixed tissues were embedded in paraffin using standard procedures and cut into 4 μm-thick sections on a Leica microtome (Buffalo Grove, IL, USA). The tissue sections were stained with hematoxylin and eosin (H&E) for evaluation of histology and with antibodies against Ki-67 (1:200, Cell Signaling #9129) (proliferation) and neuroendocrine markers synaptophysin (SYP; 1:200, Sigma-Aldrich, St. Louis, MO, USA, #336A-75), forkhead box A2 (FOXA2; 1:200, Abcam, Waltham, MA, USA, #ab108422) and insulinoma-associated protein 1 (INSM1;1:200, Santa Cruz Biotechnology, Dallas, TX, USA, #sc-271408). The stained sections were imaged with a Nikon 6D microscope under bright-field illumination using a 40× power objective, yielding a 0.22 μm in-plane resolution. Image analysis was conducted using QuPath [35]. H&E- and Ki-67-stained sections were quantified to extract cellularity (number of nuclei per mm^2^) and proliferation (% of positively stained cells), respectively.

### 2.9. Micro-Computed Tomography (CT) Image Acquisition and Analysis

Mice bearing tumors in bone were imaged using the MIlabs VECTor4/CT system. Full-body scans were acquired at 40 μm isotropic resolution. Images were viewed and rendered for presentation after thresholding using the open-source AMIDE software (Mac OS versions 10.6-10.9) [36].

### 2.10. RNA-Sequencing (RNA-Seq)

Total RNA was isolated from flash-frozen tissues using an AllPrep DNA/RNA mini kit (Qiagen, Hilden, Germany), and integrity was confirmed using an Agilent Bioanalyzer (Agilent Technologies, Santa Clara, CA, USA). Preparation of RNA library and transcriptome sequencing were performed by Novogene Corporation Inc. (Sacramento, CA, USA) using paired-end reads up to 150 bp. Sequencing reads were aligned to the reference genome using STAR version 2.5 to count the number of reads per gene while mapping. The counts coincide with those produced by htseq-count (v0.6.1) with default parameters. Fragments per kilobase million (FPKM) of each gene were calculated based on the length of the gene and read count mapped to this gene. Differential gene analysis was performed using DESeq2 (v1.14.1). The resulting *p*-values were adjusted using Benjamini and Hochberg’s approach for controlling the False Discovery Rate (FDR). Genes with an adjusted *p*-value < 0.05 found by DESeq2 were assigned as differentially expressed. Correlation was determined using the cor.test function in R with options set alternative = “greater” and method = “Spearman”. To identify the correlation between differences, we clustered different samples using expression level FPKM to see the correlation using hierarchical clustering distance method with the function of heatmap, SOM (Self-organization mapping), and kmeans using silhouette coefficient to adapt the optimal classification with default parameter in R.

### 2.11. Statistical Analysis

Data are presented as mean ± standard error of the mean. Each dot in the bar graphs corresponds to a measurement from one animal. A one-way ANOVA with post hoc tests assessed differences among relevant groups. Multiple comparisons were corrected by controlling for FDR (*q* < 0.05) using the two-stage step-up method of Benjamin, Krieger, and Yekutieli. Correlation analyses were performed using Pearson correlation tests. *p*-values < 0.05 were considered statistically significant. All statistical tests were performed using PRISM (GraphPad, Boston, MA, USA).

## 3. Results

### 3.1. Growth Characteristics of SCNC PDX Propagated Under the Renal Capsule

The three PDXs selected for our study are validated models of SCNC. LuCaP 93, derived from tissue obtained by transurethral resection of the prostate, has molecular characteristics typical of SCNC, including the loss of Rb1, p53, and PTEN [21]. The expression of the AR, prostate-specific antigen (PSA), PSMA, ERG, and alpha-methylacyl-CoA-racemase (AMACR) is absent, while the neuroendocrine markers chromogranin A and synaptophysin (SYN) are present. A classic transcriptional signature of the SCNC subtype of PCa is expressed by LuCaP 93 [37]. LTL352 was derived from a metastasis to the urethra. Proteomic as well as transcriptomic signatures of the LTL352 cluster with other SCNC PDXs [38]. LTL610 is less characterized than the other PDXs. Derived from a prostate neuroendocrine cancer, this PDX does not express the AR or PSA and is positive for SYN. All PDXs have small cell histology.

**Figure 1:**
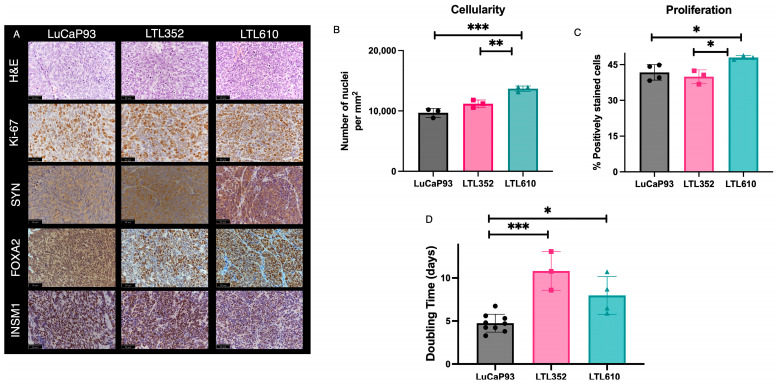
Characterization of SCNC PDX in the subrenal capsule site. (**A**) IHC staining of representative tumors including H&E, Ki-67, SYN, FOXA2, and INSM1 (40× magnification). The inset black line scale bar denotes 50 microns. Bar plots showing (**B**) the number of cells (nuclei) per mm^2^ area, (**C**) the percentage of cells staining positive for Ki-67, and (**D**) tumor doubling times of the PDX. (Note: Data are represented as mean ± SE. Significance is shown as *p* values. * *p* < 0.05, ** *p* < 0.01 and *** *p* < 0.001).

At its institution of origin (University of Washington), LuCaP 93 is propagated subcutaneously in Nu/Nu (NU-Foxn1nu) or CB-17 SCID (CB17/Icr- Prkdcscid/IcrCrl) mice [21]. LTL352 and LTL610 are routinely maintained at the Living Tumour Laboratory (Vancouver Prostate Centre) in the subrenal capsule (SRC) site in NOD-SCID mice (www.livingtumorlab.com). Here, we characterized the three PDXs implanted at the SRC site in male NOD SCID gamma (NSG) mice. Whether implanted from cryopreserved or freshly harvested specimens, the tumor take rate of tissues implanted under the renal capsule was 100% for all three PDXs (Supplementary Table S1). STR profiling of LuCaP 93 confirmed the identity as previously reported [21] (Supplementary Table S2). No prior STR profiles were available for LTL352 and LTL610, but the profile of each was unique and not matched to any other profiles in the databases (Supplementary Table S2). Immunohistology of representative tumors from each PDX is shown in Figure 1A. All three PDXs stained positive for the neuroendocrine markers SYN, FOXA2, and INSM1. LTL610 was significantly more cellular than LuCaP 93 and LTL352 (quantification of nuclei per mm^2^ in H&E-stained sections; Figure 1B) and more proliferative (percentage of Ki-67-stained nuclei per total nuclei; Figure 1C). LuCaP 93 tumors grew significantly faster with a doubling time of 5 ± 1.5 days relative to LTL352, the slowest growing with 11 ± 1.8 days, and LTL610 with 8 ± 1.8 days (Figure 1D).

### 3.2. MRI and Glycolytic Features of PDXs Propagated Under the Renal Capsule

Supplementary Figure S1A shows representative ADC, urea AUC, and k_PL_ parametric maps overlaid on T_2_-weighted proton images of tumors of each of the PDXs. All PDXs had similar mean ADC values (0.91–0.97 10^−3^ mm^2^/s) (Supplementary Figure S1B). Perfusion was evaluated by HP ^13^C-urea MRI. Urea_AUC_ (Supplementary Figure S1C) indicated that LuCaP 93 (0.77 ± 0.1 A.U.) had significantly higher perfusion compared to LTL610 (0.3 ± 0.1 A.U.) and LTL352 (0.45 ± 0.17 A.U.). The glycolytic rate (conversion of pyruvate to lactate, k_PL_), measured by HP [1-^13^C]pyruvate MRI, was significantly higher in LTL610 (0.095 ± 0.01 s^−1^) than in LuCaP 93 (0.05 ± 0.01 s^−1^) and LTL352 (0.06 ± 0.02 s^−1^) (Supplementary Figure S1D). Elevated glycolysis in LTL610 did not appear to be due to higher activity of LDH, the enzyme that converts pyruvate to lactate, as there was no significant difference between the three PDXs (Supplementary Figure S1E). The mean NAD+/NADH ratio was not significantly different between the three PDX owing to the large intra-PDX variation (Supplementary Figure S1F). Correlation analysis of tumor volumes and MRI parameters (Supplementary Figure S2, top row) showed that all three parameters (ADC, urea_AUC_, and k_PL_) were independent of tumor volume for LuCaP 93 and LTL610. However, tumor volumes of LTL352 negatively correlated with urea_AUC_, with large tumors having a lower urea_AUC_ (*p* = 0.004, R^2^ = –0.98), indicating that perfusion was restricted in large LTL352 tumors under the renal capsule. Additionally, k_PL_ and ADC were positively correlated in LTL352 tumors (*p* = 0.005, R^2^ = 0.98).

### 3.3. Growth Characteristics of SCNC PDXs Propagated in Bone

**Figure 2:**
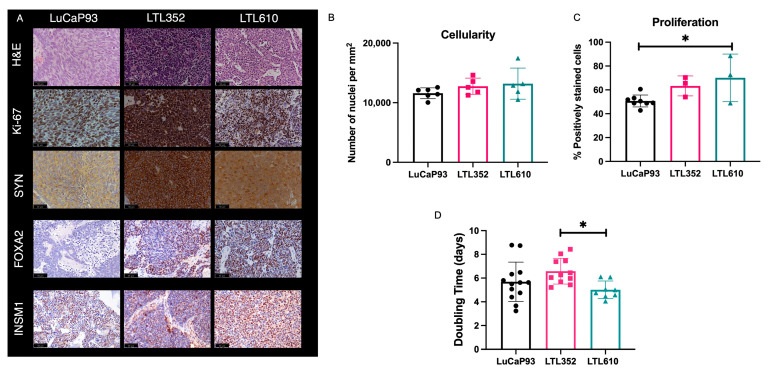
Characterization of SCNC PDXs in bone. (**A**) IHC staining of representative tumors including H&E, Ki-67, SYN, FOXA2, and INSM1) (40× magnification). The inset black line scale bar denotes 50 microns. Bar plots showing (**B**) the number of cells (nuclei) per mm^2^ area, (**C**) the percentage of cells staining positive for Ki-67, and (**D**) tumor doubling times of the PDX. (Note: Data are represented as mean ± SE. Significance is shown as *p* values. * *p* < 0.05).

**Figure 3:**
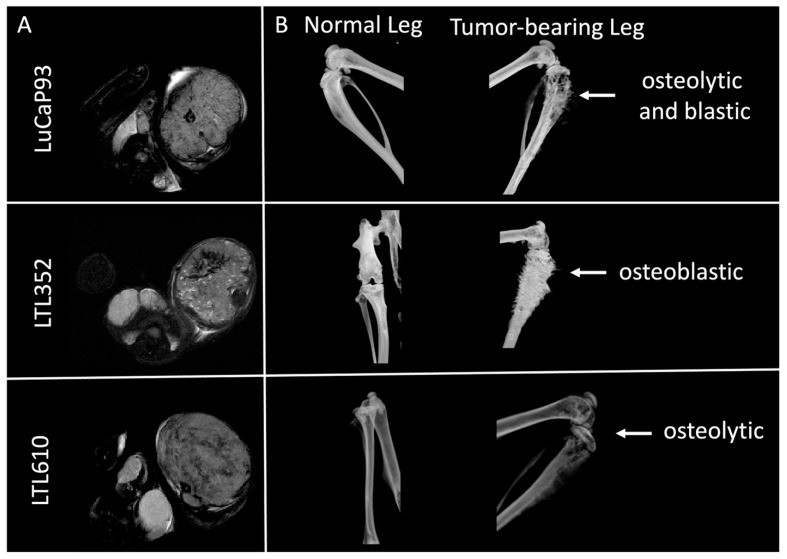
Representative MRI and µCT images of LuCaP 93, LTL352, and LTL610 tumors in bone. (**A**) T_2_-weighted images of the tumors in axial orientation. The tumors are visible as a mass in the left leg. (**B**) µCT images of the normal and tumor-bearing legs.

Single cells isolated from PDXs propagated under the renal capsule were implanted into the tibiae of male NSG mice. The tumor take rate at this site was 100% for LTL352 and LTL610 and 45% for LuCaP 93 (Supplementary Table S1). All three PDXs were positive for the neuroendocrine markers SYN, FOXA2, and INSM1 (Figure 2A). There was no significant difference in cellularity among the PDXs as judged by quantitative analysis of H&E-stained tumors (Figure 2B). Ki-67 staining showed that LTL610 (70% ± 20% positive) was significantly more proliferative than LuCaP 93 (51% ± 5%), while LTL352 had moderate Ki-67 staining (63% ± 8%) (Figure 2C). LTL352 was the slowest growing of the three PDXs (tumor doubling time 6.6 ± 0.3 days), and LTL610 was the fastest (5 ± 0.3 days); LuCaP 93 tumors doubled every 5.7 ± 0.5 days (Figure 2D). Tumor-bearing tibiae were imaged by μCT to characterize the interaction of the SCNC PDX with bone (Figure 3). Prior work by Nguyen et al. denoted LuCaP 93 tumors as purely osteoblastic [21]. However, we observed that LuCaP 93 had a mixed phenotype with both osteolytic and osteoblastic features. The phenotypes of LTL352 and LTL610 in bone were not previously reported, and we found that LTL352 was osteoblastic while LTL610 was osteolytic.

### 3.4. MRI and Glycolytic Features of PDXs Propagated in Bone

Supplementary Figure S3A shows representative ADC and k_PL_ parametric maps overlaid on T_2_-weighted proton images for LTL352 and LTL610 tumors in bone. LTL352 had a significantly (*p* = 0.0029) higher mean ADC (0.92 ± 0.05 × 10^−3^ mm^2^/s) than LTL610 (0.69 ± 0.02 × 10^−3^ mm^2^/s), while the mean ADC of LuCaP 93 tumors was 0.78 ± 0.06 × 10^−3^ mm^2^/s (Supplementary Figure S3B). The urea_AUC_ (Supplementary Figure S3C) of LTL352 (3 ± 0.2 A.U.) was significantly higher than LuCaP 93 (0.7 ± 0.2 A.U., *p* = 0.0002) and LTL610 (1.5 ± 0.3 A.U., *p* = 0.0019). The glycolytic rate of LTL352 (k_PL_ = 0.08 ± 0.01 s^−1^) was significantly (*p* = 0.0081) lower than that of LTL610 (0.13 ± 0.01 s^−1^) and lower than LuCaP 93 (0.12 ± 0.01 s^−1^), albeit not significantly (*p* = 0.0967) (Supplementary Figure S3D). Neither LDH activity (V_max_ 0.3–0.6 μM NADH/min/mg protein) nor NAD+/NADH ratio was significantly different between the PDXs (Supplementary Figure S3E,F). Correlation analysis of tumor volumes and MRI parameters showed that all three parameters (ADC, urea_AUC_, and k_PL_) were independent of the tumor volumes for all PDXs propagated in bone (Supplementary Figure S2, middle row). A significant correlation was found in LuCaP 93 tumors between urea_AUC_ and ADC (r = 1, *p* = 0.012). Upon combining all three PDXs together, the correlation between urea_AUC_ and ADC was significant, with r = 0.72 and *p* = 0.003.

### 3.5. Growth Characteristics of SCNC PDXs Propagated in Liver

**Figure 4:**
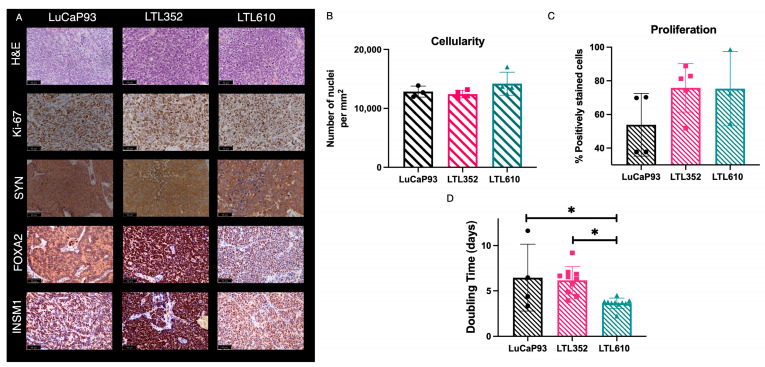
Characterization of SCNC PDX in liver. (**A**) IHC staining of representative tumors including H&E, Ki-67, SYN, FOXA2, and INSM1) (40x magnification). The inset black line scale bar denotes 50 microns. Bar plots showing (**B**) the number of cells (nuclei) per mm^2^ area, (**C**) the percentage of cells staining positive for Ki-67, and (**D**) tumor doubling times of the PDX. (Note: Data are represented as mean ± SE. Significance is shown as *p* values. * *p* < 0.05).

Single cells from PDXs propagated under the renal capsule were isolated and implanted into the liver of male NSG mice. Tumor take rate was 100% for LTL610, 71% for LTL352, and 66% for LuCaP 93 (Supplementary Table S1). All three PDXs grown in the liver stained positive for the SCNC markers SYN, FOXA2, and INSM1 (Figure 4A). No significant differences in cellularity (Figure 4B) or proliferation (Figure 4C) were noted among the PDXs. As in bone, LTL610 was the fastest growing of the PDXs in liver [tumor doubling time 3.6 ± 0.2 days relative to LuCaP 93 (6.5 ± 1.8 days) and LTL352 (6.4 ± 0.7 days)] (Figure 4D).

### 3.6. MRI and Glycolytic Features of SCNC PDXs Propagated in Liver

Supplementary Figure S4A shows representative ADC and k_PL_ parametric maps overlaid on T_2_-weighted proton images for PDXs in the liver. The mean ADC for LTL610 (1.3 ± 0.13 10^−3^ mm^2^/s) was significantly higher (*p* = 0.0314) than LTL352 (0.9 ± 0.05 × 10^−3^ mm^2^/s), while the ADC for LuCaP 93 was similar (1 ± 0.08 × 10^−3^ mm^2^/s) to that of LTL352 (Supplementary Figure S4B). All three PDXs had a similar mean urea_AUC_ (Supplementary Figure S4C). The glycolytic rate of LTL352 (k_PL_ = 0.05 ± 0.009 s^−1^) was significantly lower than that of LTL610 (0.126 ± 0.016 s^−1^, *p* = 0.0316) and LuCaP 93 (0.128 ± 0.021 s^−1^, *p* = 0.0233) (Supplementary Figure S4D). LTL610 tumors in the liver had significantly higher LDH activity (V_max_ 0.72 ± 0.02 uM NADH/min/mg of protein) than LuCaP 93 (0.59 ± 0.01 uM NADH/min/mg of protein, *p* = 0.0491) and LTL352 (0.48 ± 0.05 uM NADH/min/mg of protein, *p* = 0.0036) (Supplementary Figure S3E). NAD+/NADH ratios for the PDXs were not significantly different (Supplementary Figure S4F). No significant correlations were observed between the tumor volume and MRI parameters (ADC, urea_AUC_, and k_PL_) or between the MRI parameters themselves for any PDX in the liver (Supplementary Figure S2, bottom row).

### 3.7. Microenvironmental Impact on Functional and Metabolic Parameters of SCNC PDXs

To evaluate changes conferred on PDXs by the microenvironment at clinically relevant sites of metastases, we compared growth characteristics and MRI features between tumors propagated in bone versus liver. The tumor take rate was similar in the liver compared to bone (average 79% compared to 82%, respectively) (Supplementary Table S1). Biomarkers of the SCNC phenotype (SYN, FOXA2, INSM1) continued to be expressed at both sites (Figure 2 and Figure 4), and the cellularity of each PDX did not differ in bone versus liver (Supplementary Figure S5A). The proliferation of all PDXs was similar at both sites (Supplementary Figure S5B). LTL610 tumors doubled in volume significantly faster in the liver compared to bone, while the other two PDXs grew at the same rate in both sites (Supplementary Figure S5C). In general, many characteristics of the PDX remained similar whether propagated in bone or liver, with the exception of the growth rate of LTL610.

**Figure 5:**
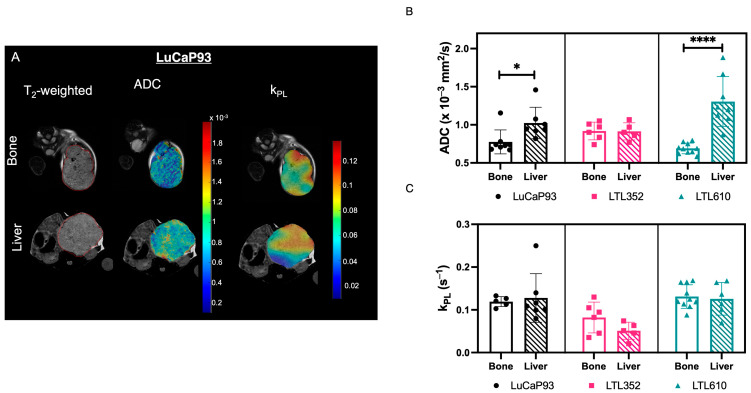
Intra PDX comparison. (**A**) Representative T_2_-weighted images of LuCaP 93 PDX implanted in bone and liver overlaid with ADC and k_pl_ maps. The tumor is delineated with a red line. Bar plots showing (**B**) ADC, and (**C**) k_pl_. (Note: Data are represented as mean ± SE. Significance is shown as *p* values. * *p* < 0.05, and **** *p* < 0.0001).

Representative ADC and k_PL_ parametric maps overlaid on T_2_-weighted proton images for LuCaP 93 in bone and liver are shown in Figure 5A. The mean ADC of both LuCaP 93 and LTL610 in the liver was significantly higher than in bone, while the mean ADC of LTL352 was similar at both sites (Figure 5B). Glycolysis (k_PL_) (Figure 5C) and the NAD+/NADH ratio (Supplementary Figure S5E) were not significantly different between bone and liver. In contrast, LDH activity was upregulated in the liver vs. the bone microenvironment in LuCaP 93 (*p* = 0.02) and LTL610 tumors (*p* = 0.0048) (Supplementary Figure S5D). Owing to the normalization of the HP urea signal to the vascular voxel in plane, it is not feasible to cross-compare the urea_AUC_ of the intrahepatic and intratibial tumors of the same PDX, as the perfusing vessel can be different for each site.

### 3.8. Steady-State Metabolism of PDXs in Bone Versus Liver

**Figure 6:**
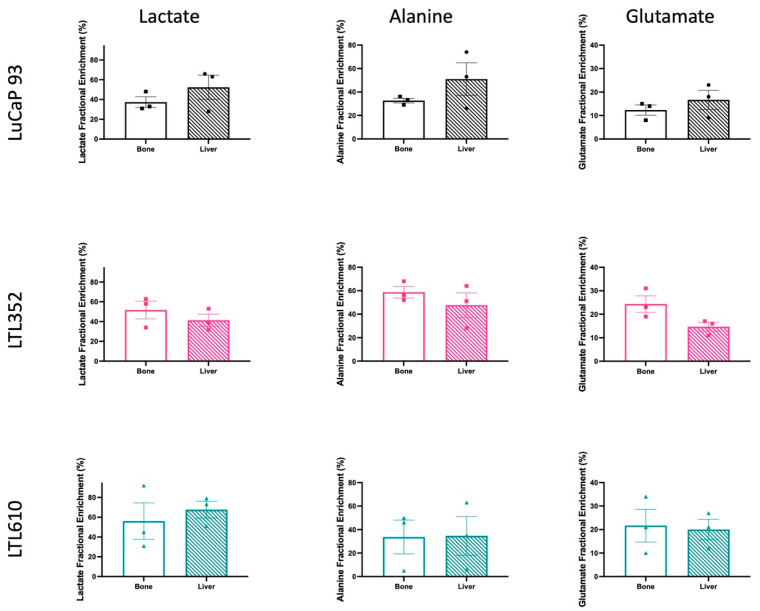
Fractional enrichment of lactate, alanine, and glutamate from glucose in the liver and bone microenvironments using stable isotopomer analysis using high-resolution magnetic resonance spectroscopy. (Note: Data are represented as mean ± SE). No significant difference is observed between sites for a given PDX.

**Figure 7:**
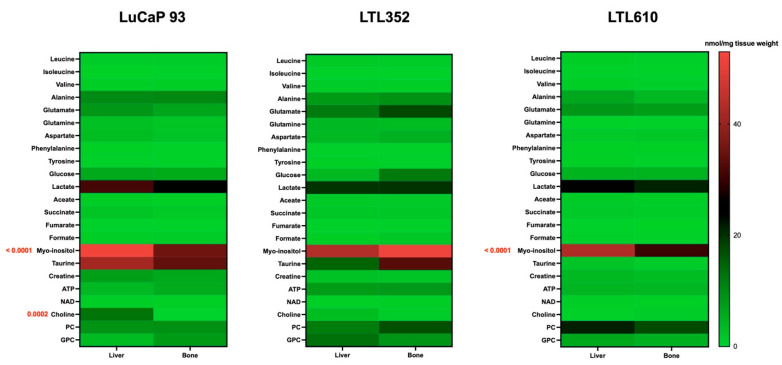
Comparison of steady state metabolite levels between LuCaP 93, LTL352, and LTL610 tumors in bone versus liver using high resolution magnetic resonance spectroscopy. Significant differences (*p*-value) between the sites are indicated in red text adjacent to the metabolites in red for each PDX.

The similar glycolytic phenotype of each PDX in bone and liver, as assessed by HP [1-^13^C]pyruvate MRI, was corroborated by high-resolution NMR metabolomic profiling of tumors labeled with ^13^C-glucose. The rate of enrichment of the lactate pool from glucose was similar in each PDX grown in bone and liver (Figure 6). Fractional enrichment of alanine and glutamate was also not significantly different between tumors in the bone versus liver. Additionally, steady-state levels of most metabolites remained unchanged irrespective of site, except for elevated myoinositol in LuCaP 93 and LTL610, and choline in LuCaP 93, in intrahepatic tumors (Figure 7).

### 3.9. Characterization of Perfusion of SCNC PDXs in Bone and Liver by Multiparametric MRI

**Figure 8:**
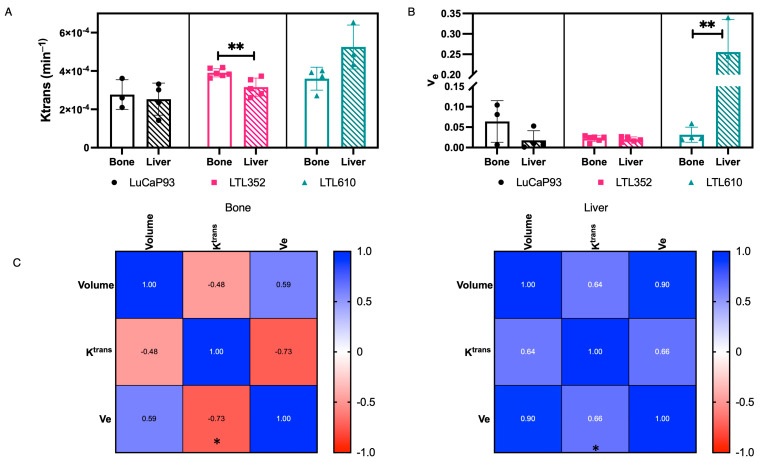
DCE-MRI characterization of PDX in bone and liver. (**A**) K^trans^, (**B**) Ve, and (**C**) correlation analysis between volume and DCE parameters. (Note: Data are represented as mean ± SE. Significance is shown as *p* values. * *p* < 0.05, ** *p* < 0.01).

Comparison between DCE-MRI parameters of each PDX in bone and liver was performed to further delineate site-specific differences in perfusion. The dynamic contrast-enhanced signal demonstrated a Type II pattern, with a rapid uptake until a steady state was reached by 2 min (Supplementary Figure S6A), and no apparent washout over 10 min in all PDXs, irrespective of site. The extravasation parameter, K^trans^, of LuCaP 93 in bone (2.8 ± 0.45 × 10^−4^ min^−1^) and liver (2.5 ± 0.42 × 10^−4^ min^−1^) was similar (Figure 8A). In comparison, the K^trans^ of LTL352 in liver (3.1 ± 0.02 × 10^−4^ min^−1^) was significantly lower (*p* = 0.0069) than in bone (3.9 ± 0.02 × 10^−4^ min^−1^), whereas the K^trans^ of LTL610 in liver (5.2 ± 0.66 × 10^−4^ min^−1^), although higher, was not statistically significant (*p* = 0.0536) than in bone (3.6 ± 0.3 × 10^−4^ min^−1^) (Figure 8A). In bone, the K^trans^ of LuCaP 93 was significantly lower than LTL352, and in liver, LTL610 was significantly higher than the other PDXs (Supplementary Figure S6B). The extracellular extravascular fraction, v_e_, was predominantly less than 10% for all PDXs across sites except for LTL610 in the liver (Figure 8B). The Vp for all the PDXs was similar between bone and liver (Supplementary Figure S6C). There was no correlation of volume to K^trans^ or v_e_, but the correlation between K^trans^ and v_e_ was significant, albeit in opposing directions in the bone (r = −0.73, *p* = 0.025) and liver (r = 0.66, *p* = 0.019) (Figure 8C).

### 3.10. Comparison of Transcriptional Landscape of PDXs in the Bone and Liver

**Figure 9:**
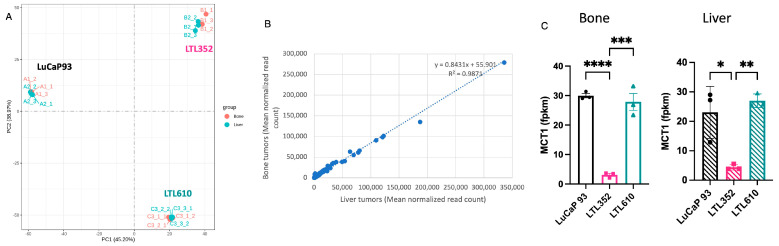
Comparison of transcriptomic profile of tumors grown in bone vs. liver. (**A**) PCA analysis of the gene expression value of each PDX in different sites (n = 3 each). (**B**) Read count congruence of bone and liver tumors. (**C**) Monocarboxylate (MCT1) transporter expression in the PDX across sites. (Note: Data are represented as mean ± SE. Significance is shown as *p* values. * *p* < 0.05, ** *p* < 0.01, and *** *p* < 0.001, **** *p* < 0.0001).

RNA-sequencing (RNA-Seq) was performed on three replicates per PDX/site. Principal component analysis of the gene expression values (FPKM) clustered all the tumors for each PDX together, irrespective of the site of growth (Figure 9A). The normalized mean read counts of individual genes of all the tumors in bone were linearly congruent with tumors in liver (Figure 9B). While LuCaP 93 and LTL352 tumors had differential expression of genes as a consequence of tumor location (38 and 147 genes, respectively), LTL610 had only 2 genes that were significantly different. Of the genes differentially expressed between the tumors of each PDX in the liver vs. bone, clusterin (CLU) was the only common gene downregulated in both LuCaP 93 (−0.969 fold, padj = 0.03) and LTL352 (−2.79 fold, padj = 1.87 × 10^−24^) in bone relative to liver (but not LTL610). Another gene, SPOCK1, was downregulated in LuCaP 93 (−0.64 fold, padj = 0.02) but upregulated in LTL352 (1.01 fold, padj = 0.006) in bone relative to liver. Recently, the impact of monocarboxylate transporters (MCTs) on k_PL_ has been reported [39]. Interestingly, MCT1 transcript levels in LTL610 were similar to those in LuCaP 93 and significantly higher than in LTL352 (Figure 9C) but did not differ by site of implantation.

## 4. Discussion

In this study, we examined the features of three SCNC PDXs maintained at an optimal and routine site of propagation (subrenal capsule) and at common sites of metastasis (bone and liver). Our in-depth metabolic and imaging studies revealed some inter-PDX differences as well as some intra-PDX differences, depending on the site of tumor propagation.

The tumor take rate was consistent and higher when PDXs were implanted under the renal capsule relative to bone or liver. The slightly diminished take rate at these sites may be due to implanting single cells into bone and liver vs. the presumably higher number of cells in tissues implanted in the kidney, or the technical challenges of injecting cells in bone and liver. Previous studies of bone engraftment of PCa cells have shown variable efficiencies ranging from 10 to 80% [21].

The growth rates of the LTL PDX are comparable to those published on the Living Tumour Laboratory website (www.livingtumorlab.com). Although cellularity, as assessed by the number of cells per area of tissue, was highest in LTL610, this was not reflected in mean ADC, which was equivalent among the PDXs. This could be due to the complex nature of ADC, representing not just cell density but also being influenced by microstructural properties such as cell size and compartmental diffusivities. Perfusion, measured by HP ^13^C-urea MRI (urea_AUC_), was significantly higher in LuCaP 93 tumors at the SRC site compared to the other PDXs, perhaps related to the cellularity (lowest of all PDXs). The only PDX for which perfusion correlated with tumor volume was LTL352, with larger tumors having significantly decreased perfusion. Incidentally, with decreased perfusion, there was no associated increase in glycolytic rate (k_PL_) or an inverse correlation of k_PL_ with volume, indicating hypoxia is not a major factor driving changes in larger LTL352 tumors. LTL610 was significantly more glycolytic than the other PDXs, as measured by the apparent rate of conversion of HP [1-^13^C]pyruvate to lactate (k_PL_). Although there was no correlation observed between LDH activity and cofactors, the trend of NAD+/NADH and k_PL_ is similar, contrary to the expectation that a tumor with higher k_PL_ would have a lower NAD+/NADH ratio. One probable explanation for this would be that the cytosolic NAD+/NADH ratio is modulated by the extracellular lactate/pyruvate ratio [40], indicating that the LTL610 tumors have higher extracellular lactate or lactate efflux relative to the other tumors. However, there was no significant difference in MCT4 (transporter predominantly responsible for efflux of lactate out of the cell) expression in LTL610 compared to the other two PDXs. The higher k_PL_ observed in LTL610 tumors is most likely due to the higher cell density (based on histology) relative to the other tumors, and not biochemically driven by LDH or its cofactor. Elevated glycolysis is consistent with the rapid proliferation, fast growth, and lower perfusion of LTL610 at the SRC site.

The bulk of our analyses focused on the comparison of tumors in bone versus liver, two major sites of clinical SCNC metastases. The ADC was significantly higher in the liver microenvironment of both LuCaP 93 as well as LTL610, and similar in both sites for LTL352. These differences in ADC suggest variations in tissue organization and composition that are induced in some but not all PDXs, depending on the site of propagation, and require advanced diffusion weighting acquisition and analysis to gain insight into the composite hindered and restricted diffusion of sub-voxel tissue structure.

Glycolysis, as reflected by k_PL_, was not altered by the site of propagation, and this was reinforced by the fractional enrichment of lactate, as measured by SIRM post-glucose labeling. However, both the LDH activity and the cofactor levels, as well as fractional enrichment of lactate, matched the glycolytic profile only in the liver. In the bone microenvironment, the LDH activity trend (although not statistically significant) was discordant with the observed k_PL_. These indicate an impact from the bone microenvironment on the measure of k_PL_ beyond the biochemical components of enzyme activity and cofactor. Additionally, pyruvate transport is mediated by MCT1, and k_PL_ may depend on levels of MCT1 as well as the activity of LDH and the NAD+/NADH ratio [41]. Interestingly, MCT1 transcript levels in the tumors matched that of the k_PL_ trend in both metastatic sites and could contribute to the apparent glycolytic rate measurement. Notably, steady-state levels of metabolites were quite similar among the PDX and were not altered by the site of propagation. Taken together, these results demonstrate that the hyperpolarized imaging metric (k_PL_) recapitulates the biochemical profile (enzyme activity and its cofactors as well as glycolytic rate) in the liver microenvironment. In the bone, the congruence between these factors is lost and is potentially influenced by the interaction of the cancer cells and the osteoclasts and osteoblasts, which needs further investigation.

Perfusion, as measured by the total AUC of HP urea signal (which is not permeability limited, and hence reflective mostly of flow), demonstrated similarity among the PDX in the liver, while in the bone, it was heterogeneous. Although the normalization was performed on different vascular regions, the computed urea_AUC_ values were similar for the LuCaP 93 tumors in both liver and bone, while the intratibial tumors were significantly better perfused than intrahepatic tumors for LTL352 and LTL610. Parameters extracted from DCE-MRI also showed that each PDX had unique characteristics between bone and liver sites. Although a direct correlation between K^trans^ and urea_AUC_ is not feasible due to the lack of both measurements from the same animal, the trend between the two parameters is similar in intratibial tumors across the PDX. In the intrahepatic tumors, LTL610 PDXs had significantly higher K^trans^, indicating increased vessel leakiness (as the urea_AUC_, which is not permeability-limited, was similar relative to the other PDXs). While no significant difference was observed amongst the PDXs in v_e_, the extracellular volume fraction in bone, an increase was observed in the LTL610 tumors in the liver relative to other PDX, in line with ADC measures. However, the congruence between ADC and v_e_ was not observed in the bone tumors. Altogether, permeability, leakiness, and extracellular volume fraction were quite variable among the PDX in the bone and liver.

Although a few consistent changes were induced in all three PDXs by propagation in the liver versus bone, several of the differential features observed in tumors in the liver are associated with more aggressive behavior. Levels of myoinositol were elevated in tumors in the liver of two PDXs. Elevated myoinositol has been associated with highly aggressive primary PCas [42]. The only gene transcript that was differentially expressed in tumors in the liver versus bone of more than one PDX was clusterin. This glycoprotein is associated with aggressive PCas [43] and is being tested as a component of a multi-panel of urinary biomarkers to classify indolent versus aggressive PCa [44]. Clusterin is also associated with resistance to chemotherapy, and an antisense oligonucleotide (custirsen) that can reduce levels of clusterin has been tested in clinical trials of ARPC [45]. Whether clusterin is preferentially overexpressed in metastases to the liver and has a role in the worse outcome of patients with cancer at this site may be an interesting avenue of investigation.

One caveat of our study is that the tumors we studied were not a consequence of spontaneous metastasis from the primary tumor, and thus, the cells that proliferated and formed tumors after direct injection into bone or liver were not subjected to the selection processes inherent in spontaneous metastasis. Comprehensive metabolic and imaging characterization of spontaneous metastases is hindered in several ways. First, many PCa PDXs do not spontaneously metastasize to the bone or liver, or do so rarely. Second, often the growth of the primary tumor necessitates termination of the study before metastases become large enough for functional imaging. Thus, intratibial and intrahepatic injection of cells is a viable option for studying the interaction of tumors with the host microenvironment.

The information provided from our study will facilitate future preclinical studies with SCNC PDX. Currently, more than 50 PDXs defined as neuroendocrine PCa exist [14]. These PDX have already provided important clinically relevant information. LuCaP 93 was included in a panel of PDX that were used to identify therapeutic vulnerabilities of SCNC, implicating BCL-2 as a target druggable by navitoclax [15]. LTL352 at the subcutaneous site was used to evaluate an aurora kinase inhibitor, to which it was very sensitive [46], although aurora kinase inhibitors have not yet proven useful to treat SCNC. Expansion of preclinical studies with SCNC PDX to encompass heterogeneity, and in fact, the heterogeneity of AR-negative ARPC, including SCNC, will be required to identify diverse imaging and treatment strategies [47]. These initial imaging studies using HP [1-^13^C] pyruvic acid clearly demonstrate the ability to measure glycolytic capacity, the dominant bioenergetic pathway in tumors, at clinically relevant sites of metastases, and the numerous factors such as enzymatic activity, cofactor availability, cellularity, and perfusion that can influence the measured rate, k_PL_. For example, based on the measured k_PL_ values of LuCaP 93 tumors in the liver (as they had the largest variance, with a mean of 0.1278 s^−1^ and standard deviation of 0.0569 s^−1^), we calculated that a 30% reduction in k_PL_ will result in an effect size Cohen’s f of 1.438. For a standardized correlation among repeated measures of 0.2, the sample size required is six for two groups and two measures per group, yielding a power of 0.98 (computed using the G*Power calculator, Version 3.1 [48]). These denote a reasonable sample size and support the feasibility of imaging trials for biomarker development of treatment response.

## 5. Conclusions

In conclusion, we demonstrated that metabolic profiles of the PDXs investigated are not impacted by the microenvironment in the bone and liver, while the physiologic measures of diffusion and perfusion were highly variable and can contribute to the measured k_PL,_ especially in the bone microenvironment. Hence, it is important to characterize tumors using a combination of HP ^13^C MRI and ^1^H multi-parametric imaging to better understand the biological basis of the MRI signals, which will be instrumental in developing imaging biomarkers of response to treatment.

## Data Availability

The original contributions presented in this study are included in the article/Supplementary Material. Further inquiries can be directed to the corresponding author.

## Supplementary Materials

The following supporting information can be downloaded at https://www.mdpi.com/article/10.3390/cancers17142385/s1. Figure S1: Characterization of MRI and metabolic features of PDX tumors in SRC site; Figure S2: Heatmap of correlation matrix of Pearson coefficients of PDX propagated in SRC, bone and liver sites; Figure S3: Characterization of MRI and metabolic features of PDX tumors in bone; Figure S4: Characterization of MRI and metabolic features of PDX in liver; Figure S5: Molecular and cellular comparisons between bone and liver for each PDX; Figure S6: Comparison of DCE parameters among PDX in bone and in liver; Table S1: Tumor take rate of LuCaP 93, LTL352 and LTL610 in subrenal capsule, liver and bone sites; Table S2: STR profiles of SCNC PDX.
