# Supplementary material for: Impact of Metastatic Microenvironment on Physiology and Metabolism of Small Cell Neuroendocrine Prostate Cancer Patient-Derived Xenografts
Source: Cancers (Basel). 2025 Jul 18;17(14):2385. doi: 10.3390/cancers17142385 (PMC12293749; doi:10.3390/cancers17142385)
Supplement: Supplementary file 1 [file cancers-17-02385-s001.zip › cancers-3708765-supplementary.pdf]

Supplementary Materials

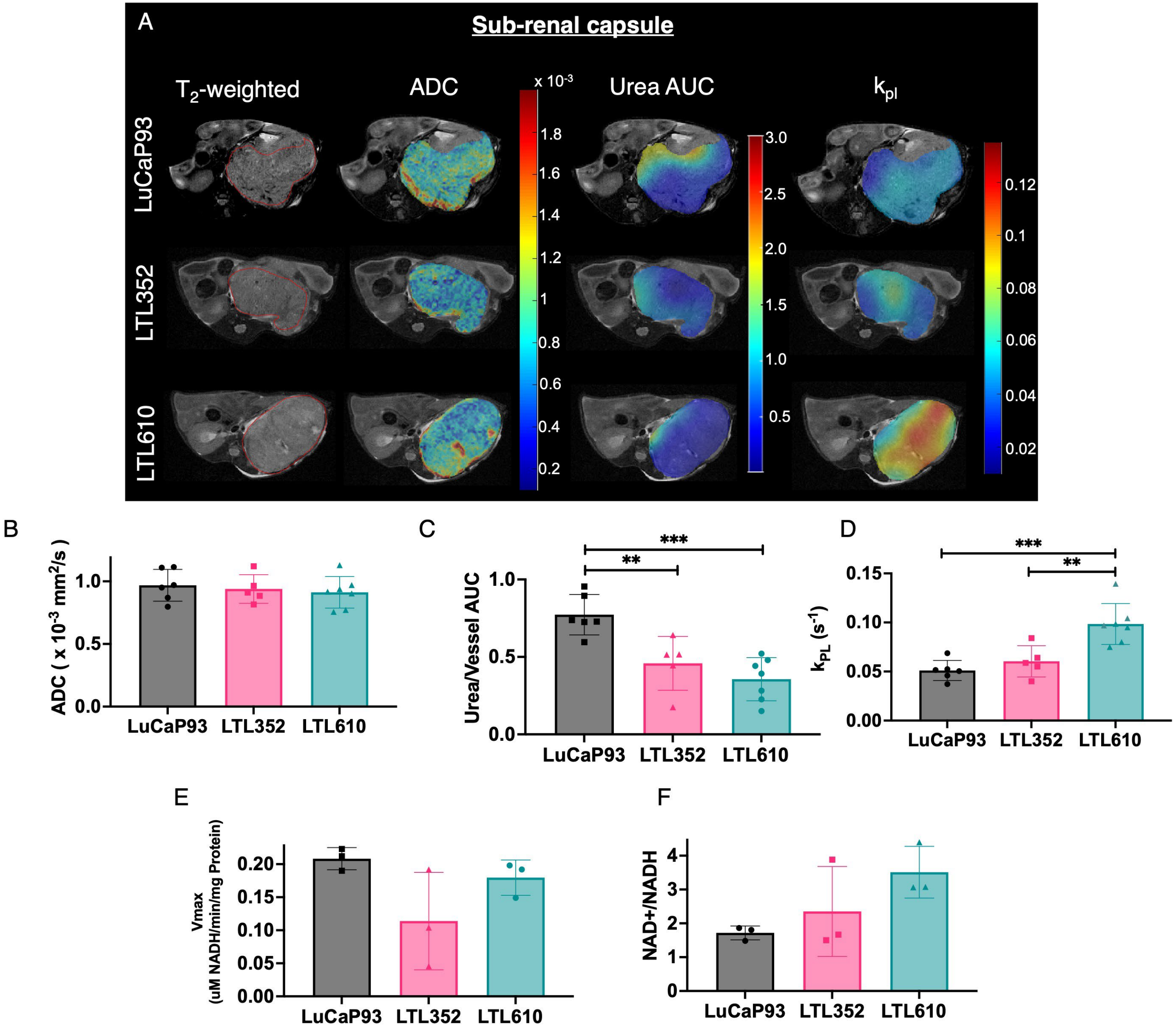

Figure S1: Characterization of MRI and metabolic features of PDX tumors in SRC site. (A) Representative T2-weighted images of PDX overlaid with ADC, ureaAUC and kpl maps. The tumor is delineated with red line. Bar plots showing (B) mean ADC, (C) ureaAUC, (D) kpl , (E) LDH activity and (F) NAD+/NADH quantification. (Note: Data are represented as mean ± SE. Significance shown as p values. **p<0.01 and ***p<0.001).

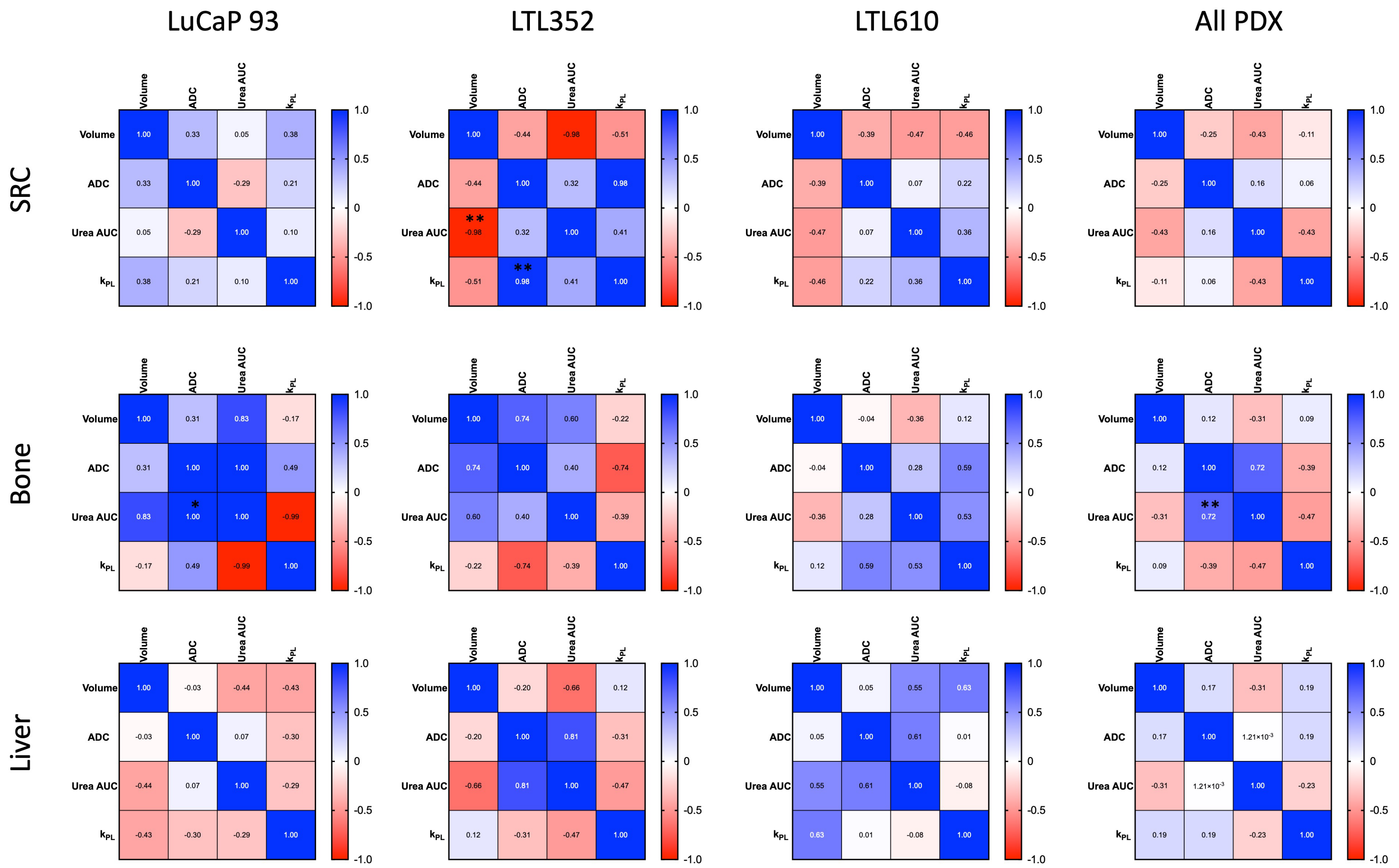

Figure S2: Heatmap of correlation matrix of Pearson coefficients of PDX propagated in SRC, bone and liver sites. * p<0.05 and ** p<0.01

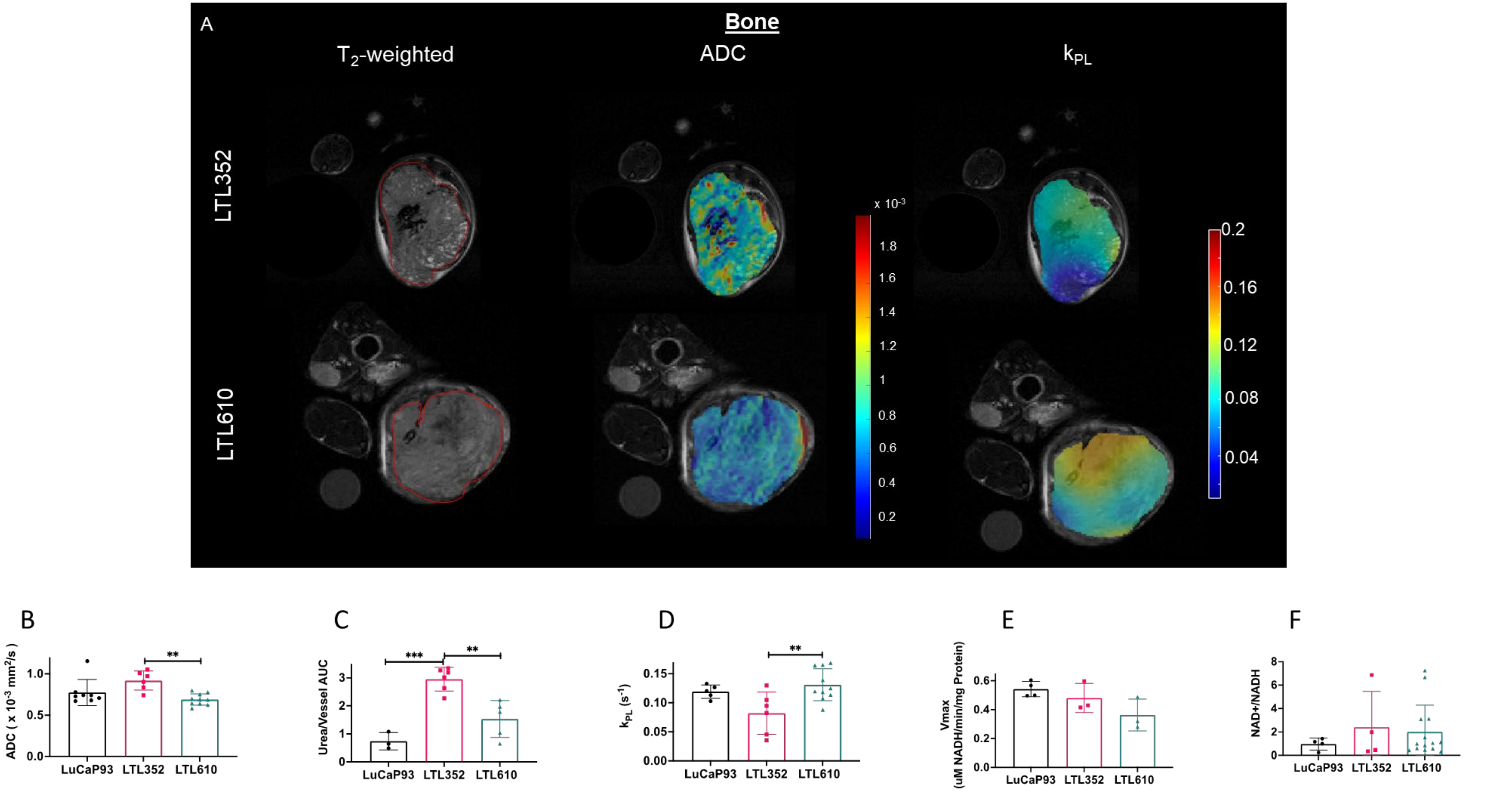

Figure S3: Characterization of MRI and metabolic features of PDX tumors in bone. (A) Representative T2-weighted images of LTL352 and LTL610 PDX overlaid with ADC and kpl maps. The tumor is delineated with red line. Bar plots of (B) mean ADC, (C) ureaAUC, (D) kpl, (E) LDH activity and (F) NAD+/NADH quantification. (Note: Data are represented as mean ± SE. Significance shown as p values. * $p<0.05$ and ** $p<0.01$, *** $p<0.001$).

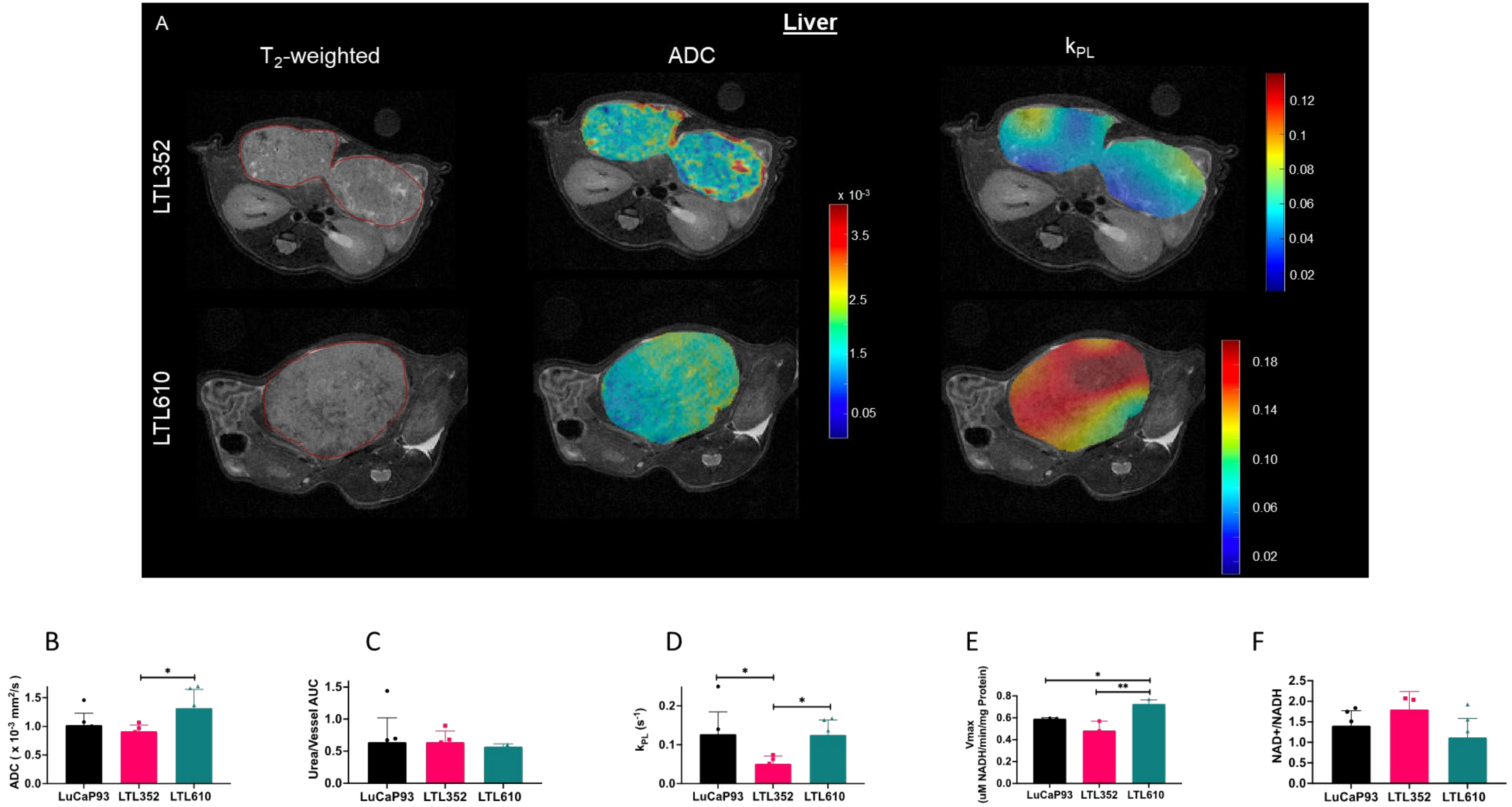

Figure S4: Characterization of MRI and metabolic features of PDX in liver. (A) Representative T2-weighted images of LTL352 and LTL610 PDX implanted in liver overlaid with ADC, and kpl maps. The tumor is delineated with red line. Bar plots of (B) mean ADC, (C) ureaAUC, (D) kpL, (E) LDH activity and (F) NAD+/NADH quantification. (Note: Data are represented as mean ± SE. Significance shown as p values. * $p<0.05$ and ** $p<0.01$).

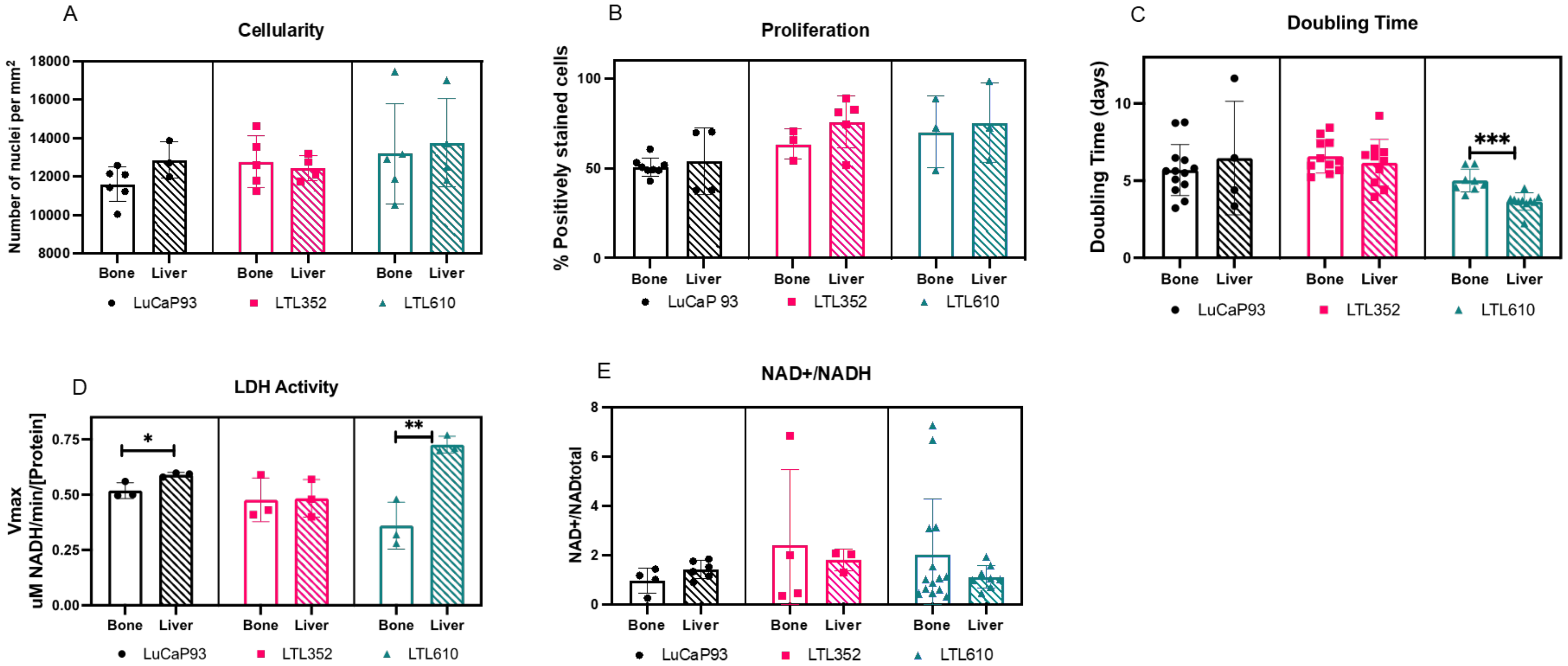

Figure S5: Molecular and cellular comparisons between bone and liver for each PDX. (A) Cellularity measured using H&E, (B) proliferation assessed by quantifying Ki67 staining, (C) doubling time, (D) calorimetric assay of LDH enzyme activity and (E) ratio of LDH cofactor nicotinamide adenine dinucleotide comparisons between bone and liver tumors of the same PDX. (Note: Data are represented as mean ± SE. Significance shown as p values. * $p<0.05$, ** $p<0.01$ and *** $p<0.001$).

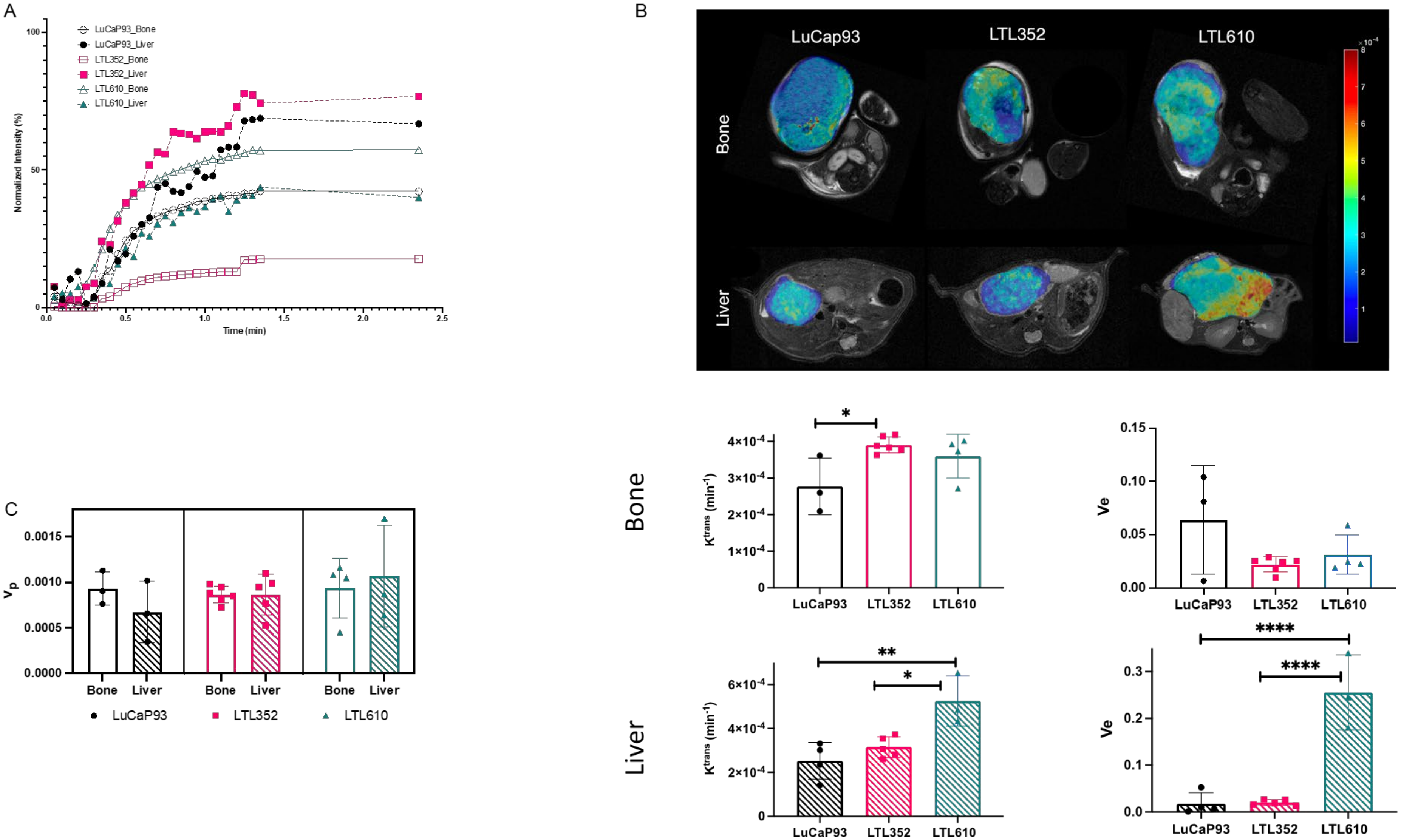

Figure S6: Comparison of DCE parameters among PDX in bone and in liver. A) Representative dynamic enhancement curve of PDX tumors. B) Representative T2-weighted images of each PDX implanted in bone and liver overlaid with Ktrans maps and quantitative comparison of Ktrans and Ve. C) Comparison of Vp between sites for a given PDX .(Note: Data are represented as mean ± SE. Significance shown as p values. * p<0.05, **p<0.01, and **** p<0.0001).

Table S1. Tumor take rate of LuCaP 93, LTL352 and LTL610 in subrenal capsule, liver and bone sites.

	LuCaP 93	LTL352	LTL610
**Subrenal Capsule**	100% (n=13, frozen)	100% (n=10 fresh and n=3 frozen)	100% (n=10 fresh and n=5 frozen)
**Bone**	45% (n=5 fresh and n=27 frozen)	100% (n=8 fresh and n=5 frozen)	100% (n=9 fresh and n=10 frozen)
**Liver**	66% (n=12, frozen)	71% (n=7 fresh and n=7 frozen)	100% (n=19 frozen)

Table S2: STR profiles of SCNC PDX.

	LuCaP 93		LTL352	LTL610
STR Locus	University of Washington	UCSF	UCSF	UCSF
TH01	8	8	9,3	9,3
D5S818	11	11	12	11,12
D13S317	10, 11	10, 11	12, 14	12
D7S820	9, 12	9, 12	9	11
D16S539	12	12	9	11
CSF1PO	11	11	10, 12	11, 12
Amelogenin	X,X	X	X,Y	X,Y
vWA	18, 20	18, 20	14, 17	16
TPOX	8	8	9, 11	8, 10
